# Evaluation of Lipid Nanoparticles as Vehicles for Optogenetic Delivery in Primary Cortical Neurons

**DOI:** 10.3390/pharmaceutics18010004

**Published:** 2025-12-19

**Authors:** José David Celdrán, Lawrence Humphreys, Maria Jose Verdú, Desirée González, Cristina Soto-Sánchez, Gema Martínez-Navarrete, Lucía Enríquez, Iván Maldonado, Idoia Gallego, Mohamed Mashal, Noha Attia, Gustavo Puras, José Luis Pedraz, Eduardo Fernández

**Affiliations:** 1 Biomedical Neuroengineering, Institute of Bioengineering (IB), University Miguel Hernández (UMH), 03020 Elche, Spain; jose.celdran.lopez@gmail.com (J.D.C.); lawrencehumphreys@hotmail.com (L.H.); maria.verdu07@goumh.umh.es (M.J.V.); dgonzalez@umh.es (D.G.); csoto@umh.es (C.S.-S.); gema.martinezn@umh.es (G.M.-N.); 2 Networking Research Centre of Bioengineering, Biomaterials and Nanomedicine (CIBER-BBN), Institute of Health Carlos III, 28029 Madrid, Spain; lucia.enriquez@ehu.eus (L.E.); ivan.maldonado@ehu.eus (I.M.); idoia.gallego@ehu.eus (I.G.); gustavo.puras@ehu.eus (G.P.); joseluis.pedraz@ehu.eus (J.L.P.); 3 NanoBioCel Research Group, Laboratory of Pharmacy and Pharmaceutical Technology, Faculty of Pharmacy, University of the Basque Country (UPV/EHU), 01006 Vitoria-Gasteiz, Spain; mashal313@yahoo.com (M.M.); noha.attia@alexmed.edu.eg (N.A.); 4 Bioaraba, NanoBioCel Group, School of Pharmacy, University of the Basque Country (UPV/EHU), 01006 Vitoria-Gasteiz, Spain; 5 Joint Research Laboratory (JRL) on Bioprinting and Advanced Pharma Development, A Joined Venture of TECNALIA, Centro de Investigación Lascaray Ikergunea, Avenida Miguel de Unamuno, 01006 Vitoria-Gasteiz, Spain

**Keywords:** lipid nanoparticles, optogenetics, lipofectamine, neuron delivery

## Abstract

**Background**: Gene therapy has experienced significant development since its origin decades ago, resulting in therapies for a wide range of diseases. In this context, optogenetics has emerged as a promising therapy for treating diseases in a precise spatiotemporal way using light. Transporting optogenetic genes to target cells is achieved using viral vectors, specifically AAV vectors. These vectors present limited cargo capacity, and a large percentage of the population carries AAV neutralizing antibodies. In this regard, lipid nanoparticles can overcome some of the previously mentioned problems of AAV vectors, making them prime candidates for optogenetic delivery. **Methods**: In this study, we evaluated their suitability for the delivery of the ChrimsonR plasmid in neurons in vitro. **Results**: In rat cortical neurons, in most of the concentrations tested, there was no reduction in several neuron morphological parameters that we measured when compared to another non-viral nanoparticle called lipofectamine. Transfection efficiency was significantly higher compared to lipofectamine in almost all treatments. Further in vitro analysis showed that electrophysiological parameters were altered, with reduced signal amplitudes; however, cell viability assays showed no decline in cell viability. **Conclusions**: These results demonstrate that lipid nanoparticles represent a promising non-viral platform for optogenetic delivery, though formulation optimization is required to achieve full functional efficacy.

## 1. Introduction

Gene therapy, a therapy consisting of adding, removing, or modifying genetic material for treating human diseases, has been remarkably developed since its origin in the 1970s [1]. As of 2025, 23 gene therapies have gained regulatory approval worldwide, addressing a broad spectrum of disorders, including CNS disorders, cancer, and cardiovascular diseases [2]. Many gene therapies have also been employed for the treatment of rare diseases, with approved therapies for Leber congenital amaurosis [3], Duchenne muscular dystrophy [4], and metachromatic leukodystrophy [5], among others, highlighting the potential of gene therapy to treat human diseases.

In this regard, optogenetics, a method which enables the control of neural activity through the use of light-activated proteins (opsins) in targeted cells [6], has emerged as a promising alternative to treat genetic diseases. Optogenetics allows the precise control of specific types of cells through the use of genetic promoters [7,8,9,10] with millisecond precision and can either activate or inhibit cells [11,12], achieving unprecedented manipulation of activity in neural circuits. Even though optogenetics is a relatively recent technology, several groups have currently registered early phase clinical trials in patients affected by retinitis pigmentosa (RP), in which they have expressed different optogenetic genes with different characteristics [13,14,15].

To carry the optogenetic genes to mammalian cells, the use of adequate vectors that can package these genes and unpack them once they are internalized by cells is essential. To date, the most used vectors in optogenetics (including all of the optogenetic clinical trials) have been the adeno-associated viruses (AAVs)-based vectors [16]. The success of AAV vectors lies in high transduction efficiency, no risk of human disease even with wild-type AAVs, no strong immunogenicity (only viral elements are the inverted terminal repeats of their genome), and long-term expression of the cargo due to the non-integration in the host chromosome [17]. However, one of the disadvantages of AAV vectors is the limited cargo capacity, providing less than 5 kb for packaging of foreign DNA [18,19], which limits the possibility of introducing complex genetic constructs into cells. Another drawback is that, depending on the serotype of the AAV, a large percentage of human beings harbor antibodies that neutralize AAV transduction (NAbs), up to 60% to certain serotypes [20], although the percentage of human beings with NAbs to certain serotypes varies depending on the geography [21], and even cross-reactivity among AAV serotypes in patients with NAbs has been reported [22].

Non-viral nanoparticle vectors, on the other hand, have some advantages compared to AAV vectors, such as poor immunogenicity (absence of viral components) and higher cargo capacity [23]. Another advantage is that they are cheaper and easier to manufacture and scale up [24]. Nanoparticles are classified into three main types: organic, polymeric, and inorganic [25]. One type of organic nanoparticles, the lipid nanoparticles (LNPs), have drawn industrial attention for their physicochemical stability, ease of preparation, and scalability [26]. In gene therapy, LNPs have been employed for delivering plasmid DNA [27,28,29,30], mRNA [31,32], and siRNA [33,34]. This genetic delivery has been transferred to different diseases, such as cancer [32,35,36], rare diseases like Fabry disease [37], hepatic diseases like liver cirrhosis [38], and even to fight viral infections, having been used in the mRNA vaccines against SARS-CoV-2 [39]. Although their capacity to deliver nucleic acids is proven, there are no reports of LNPs delivering optogenetic genes into neural cells.

In this work, to the best of our knowledge, we report the first combination of LNP-based genetic delivery with an optogenetic plasmid into in vitro cortical cells. More precisely, we deliver a plasmid which codifies for the ChrimsonR protein, a red-shifted opsin (activated at 590 nm) with high cellular trafficking and fast-kinetics [40]. Our primary goal was to assess the feasibility of this strategy for neuronal photosensitization in optogenetic therapies. To this end, we characterized the morphology, transfection efficiency, electrophysiology, and cell viability of rat cortical cells transfected with one formulation of LNPs combined with the ChrimsonR plasmid.

## 2. Materials and Methods

### 2.1. Elaboration of Lipid Nanoparticles

Lipid nanoparticles (LNPs) containing plasmid DNA (pChrimsonR) were prepared by microfluidic mixing of an ethanol organic phase containing lipidic components (Lin-KC2-DMA, 1,2-dipalmitoyl-sn-glycero-3-phosphocholine (DPPC), cholesterol, and 1,2-Dimyristoyl-rac-glycero-3-methoxypolyethylene glycol-2000 (DMG-PEG2K) in a molar ratio of 50:10:38.5:1.5, respectively) and an aqueous solution of sodium acetate buffer (pH 4.5) containing genetic material using a NanoAssemblr Ignite (Precision Nanosystems, Vancouver, BC, Canada). LNPs were formulated at an aqueous/organic flow rate ratio (FRR) of 3/1, and a N/P ratio of 6 with a total flow rate of 6 mL/min. Then, formulations were dialyzed at 4 °C using a Slide-A-Lyzer Dialysis Cassette 10K MWCO (Thermo Fisher Scientific, Waltham, MA, USA) against PBS (pH 7.4). To achieve the desired concentration of LNP formulations, samples were subjected to centrifugation using a 10K MWCO Amicon^®^ Ultra Centrifugal filter (Merck KGaA, Darmstadt, Germany) at 2000× *g* and 4 °C until the target volume was obtained.

The pChrimsonR plasmid embedded into the LNPs was obtained from Edward Boyden (Addgene plasmid # 59169) and subsequently amplified and purified using the Qiagen endotoxin-free plasmid purification Maxi-prep kit (Qiagen, Valencia, CA, USA), following the manufacturer’s protocol. The concentration and purity of the resulting plasmid was determined using a NanoDrop™ 2000 Spectrophotometer (Thermo Fisher Scientific, Waltham, MA, USA).

### 2.2. Physicochemical Characterization of LNPs

LNP formulations were characterized for particle size diameter, polydispersity index (PDI), and zeta potential by dynamic light scattering (DLS) using a Zetasizer Nano ZS (Malvern Panalytical, Worcestershire, UK). Encapsulation efficiency was quantified using Quant-it PicoGreen DNA Assay Kit (Thermo Fisher Scientific) according to the manufacturer’s protocol applying the following Formula (1):

(1) $$Encapsulation\;efficiency\left(\%\right)=\frac{F_{total}-F_{unencapsulated}}{F_{total}}\times 100$$

The morphology of the resulting LNPs was examined by transmission electron microscopy (TEM) TECNAI G2 20 TWIN (FEI, Eindhoven, The Netherlands). Briefly, 5 μL of each sample was applied onto glow-discharged carbon coated grids for 60 s. Excess liquid was removed by blotting with filter paper and stained with 2% uranyl acetate for 60 s. Samples were visualized using aTecnai G2 20 Twin microscope (FEI, Eindhoven, The Netherlands) operated at an accelerating voltage of 200 keV in bright-field mode. Digital images were acquired with an Olympus SIS Morada camera (Olympus, Tokyo, Japan).

### 2.3. Animal Models

For in vitro experiments, E16-E19 rat embryos (Wistar) were used for the extraction of primary neuronal cells. All procedures complied with Spanish RD 53/2013 and European Directive 2010/63/EU on the protection of animals used for scientific purposes. Experimental protocols were reviewed and approved by the Miguel Hernández University Standing Committee for Animal Use in the Laboratory under authorization codes UMH.IB.EFJ.02.18 for rats (approval date: 10 January 2019).

### 2.4. Primary Neuronal Cell Extraction and Culture

Primary neuronal cells were isolated from embryonic Wistar rat brains and kept in DMEM (GIBCO^®^, Thermofisher Scientific, Waltham, MA, USA) containing 10% fetal bovine serum (FBS; Biowest^®^, Nuaillé, Pays de la Loire, France) during the extraction process. Next, we replaced DMEM with 10% FBS with FBS-free DMEM to allow chemical dissociation. For chemical dissociation, cells were treated with trypsin 0.05% and incubated at 37 °C. After determining cell density in a hemocytometer, cell suspensions were adjusted according to the requirements of each experimental analysis. For morphological, transfection efficiency, and electrophysiological evaluation of cortical neurons transfected with LNPs, cells were resuspended in Neurobasal™(GIBCO^®^) medium supplemented with FBS, B27, GlutaMAX, and penicillin–streptomycin (GIBCO^®^), and seeded at 1.5 × 10^5^ cells per well on glass coverslips in 24-well plates. Cultures were maintained in an incubator at 37 °C and 5% CO_2_. For viability assays, cells were resuspended in the same Neurobasal™ supplemented medium and seeded at 1.5 × 10^4^ cells per well in 96-well plates.

### 2.5. In Vitro Transfection in Primary Neuronal Cell Culture

Cells maintained in Neurobasal™ medium (Thermo Fisher Scientific, Grand Island, NY, USA) were seeded into 24-well plates and cultured for either 21–28 or 7–11 days in vitro (DIV) prior to transfection. Transfections were performed with a range of concentrations of LNPs from 0.25 µg to 2 µg with increments of 0.25 µg. Before application, LNP concentrations were incubated for 5 min in serum-free OptiMEM solution (GIBCO^®^). Cells were then exposed to LNPs for 4 h at 37 °C in the incubator, removing the transfection medium and replacing it with fresh Neurobasal™ medium afterwards. Lipofectamine™ 2000 (Invitrogen, Carlsbad, CA, USA) at 1.25 µg served as a positive control. The same procedure was applied to cells seeded in 96-well plates, except the plasmid concentration in each well was five times smaller (e.g., if one well had 1.25 µg in a 24-well plate, it would have been 0.25 µg in a well from a 96-well plate).

### 2.6. Morphological Evaluation and Transfection Efficiency Analysis of Transfected Cultured Cortical Neurons

A total of 24 h after the LNPs exposure, Neurobasal™ medium was removed from 24-well plates and cells were fixed with 4% paraformaldehyde (PFA; Sigma-Aldrich, Burlington, MA, USA) for 20 min. Samples were then washed twice with phosphate buffer (PB; Sigma-Aldrich, Burlington, MA, USA) concentrated at 0.1 M for 10 min each. Permeabilization and blocking were performed simultaneously by incubating cells with PB 0.1 M with 0.5% Triton X-100 and 10% FBS 1 h. Cells at 21–28 DIV were incubated overnight at 4 °C with anti-rabbit NeuN monoclonal antibody (1 mg/mL, 1:500 dilution, Thermo Fisher Scientific, Waltham, MA, USA), while cells at 7–11 DIV were processed under the same conditions with anti-rabbit MAP2 monoclonal antibody (1 mg/mL, 1:500 dilution, Millipore, Burlington, MA, USA). After two additional washes with PB 0.1 M for 10 min each, cells were incubated for 1 h with AlexaFluor^®^ 488 donkey anti-rabbit secondary antibody (2 mg/mL, 1:1000 dilution, Invitrogen, Themofisher). Cell nuclei were stained with Hoechst 33342 (Sigma-Aldrich, Burlington, MA, USA) for 10 min. Coverslips were removed from wells with tweezers and mounted onto slides using Mowiol^®^ 4-88 (Sigma-Aldrich, Burlington, MA, USA). Fluorescence images were acquired using a Zeiss Axio Observer fluorescence microscopy with Apotome illumination system (Zeiss Axiobserver, Jena, Germany).

After the acquisition of fluorescence images, morphological analysis was carried out with the plugin NeuronJ from Fiji app (ImageJ, version 2.9.0; National Institutes of Health, Bethesda, MD, USA). The parameters analyzed in rat cortical neurons included the number of dentrites, the branching points, and the total length of all dendrites.

Cells for transfection efficiency assays were treated as described above. For quantitative analysis of transfected cells, 20 random images of cells from each coverslip were taken and quantification was performed with a CellProfiler™ (version 4.2.1; Broad Institute, Cambridge, MA, USA) custom script.

### 2.7. Electrophysiological Recordings

At 21–28 DIV, transfected coverslips were removed from wells using tweezers and transferred to an extracellular medium containing (in mM): 136 NaCl, 2.5 KCl, 10 HEPES, 10 Glucose, 2 CaCl_2_, 1.3 MgCl (pH = 7.3). Patch clamp pipettes were obtained from borosilicate glass capillaries (1B150F-4, World Precision Instruments, Sarasota, FL, USA) with a resistance of 3–5 MΩ using a P97 puller (Sutter Instrument Co., Novato, CA, USA). These glass capillaries were filled with an intracellular medium containing (in mM): 130 K^+^-gluconate, 10 NaCl, 1 EGTA, 0.133 CaCl_2_, 2 MgCl_2_, 10 HEPES, 3.5 MgATP, 1 NaGTP (pH = 7.3). Targeted cells were identified under fluorescence guidance and patched with a recording electrode. Whole-cell recordings were performed using a HEKA EPC 10 USB double patch clamp amplifier (Harvard Bioscience, Inc., Holliston, MA, USA). Photocurrents were measured in voltage-clamp mode at a holding potential of −60 mV, and membrane potential was monitored under current-clamp conditions during light stimulation. Cell stimulation was performed using a monochromatic light source (pE-300 ultra, CoolLED, Andover, UK) during electrophysiological recordings. To assess photoresponses in ChrimsonR positive cells, we applied two 5 ms light pulses at 590 nm (20 W, 1 s interval).

### 2.8. Cell Viability Analysis

Cell viability in primary neuronal cell cultures at both 21–28 and 7–11 DIV was assessed after LNP exposure using the tetrazolium salt 3-[4,5-dimethylthiazol-2-yl]-2,5-diphenyl tetrazolium bromide (MTT; Sigma-Aldrich, Burlington, MA, USA) colorimetric assay. Measurements were performed at 24 h (21–28 DIV), 48 h (7–11 DIV), and 96 h (7–11 DIV). Absorbance values were recorded at 570 nm using a 2100-C microplate reader (Neuvar Inc., Palo Alto, CA, USA) following the manufacturer’s instructions. Untreated cells maintained in OptiMEM served as positive controls, and all results were normalized to these controls.

### 2.9. Statistical Analysis

For assessing normality in the data, Kolmogorov–Smirnov and Shapiro–Wilk tests were performed. We evaluated the differences between two groups using a Mann–Whitney U-test in non-parametric conditions, and a Welch’s test in parametric conditions. Results shown in bar graphs are presented as mean ± SD, whereas the results shown in boxplot graphs are presented as median (central line), interquartile range (box), and total range (whiskers). Statistical significance was set at *p*-value < 0.05. GraphPad Prism 8.0 statistical package was used to perform the analyses.

## 3. Results

### 3.1. Physicochemical Characterization of KC2-ChrimsonR LNPs

**Figure 1:**
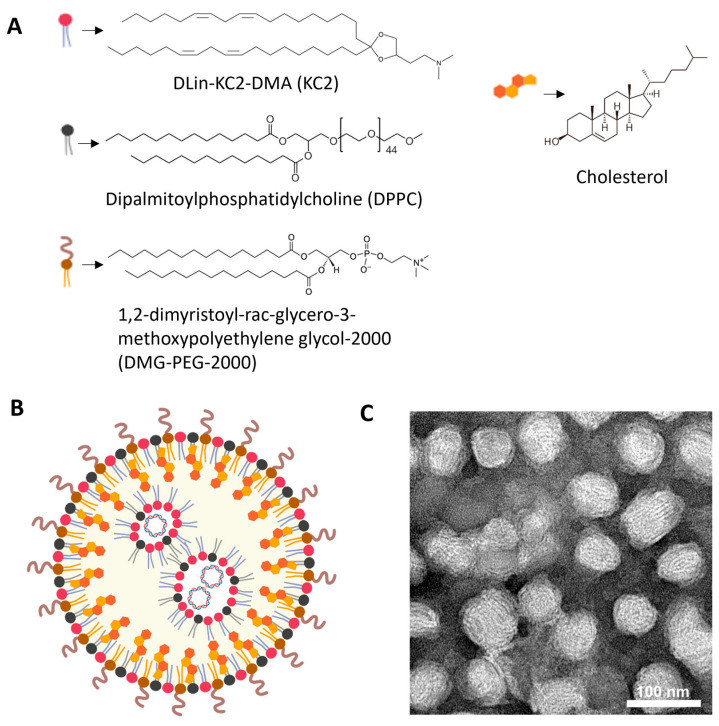
Overview of KC2-ChrimsonR LNPs. (**A**) Chemical components of KC2-ChrimsonR LNPs. (**B**) General scheme of the disposition of components in a KC2-ChrimsonR LNP with ChrimsonR plasmid encapsulated. (**C**) TEM image of KC2-ChrimsonR LNPs. Scale bar: 100 nm. Image (**B**) created in BioRender. Celdran, J (2025) https://BioRender.com/xh1gsku.

KC2-ChrimsonR LNPs contained DLin-KC2-DMA cationic lipid (KC2, 50%), dipalmitoylphosphatidylcholine (DPPC, 10%), cholesterol (38.5%), and 1,2-dimyristoyl-rac-glycero-3-methoxypolyethylene glycol-2000 (DMG-PEG-2000, 1.5%), and the ChrimsonR plasmid (Supplementary Figure S1), as depicted in Figure 1A,B. DLin-KC2-DMA ionizable cationic lipid has endosomal escape properties mainly due to its specific molecular shape, which promotes endosomal membrane disruption and payload release into the cytoplasm. Also, its pKa value (6.7) contributes to disrupting the endosome membranes, being neutral at physiological values (around 7.4) and positively charged in the endosome, where the pH gradually decreases to as low as 4.5–5.5 in late endosomes/lysosomes [41]. When observed under TEM, KC2-ChrimsonR LNPs showed a clear spherical morphology (Figure 1C, Supplementary Figure S2). The physicochemical analysis of this formulation revealed that the mean particle size was 82.295 ± 7.687 nm, the PDI was 0.138 ± 0.053, the zeta potential was −7.311 ± 7.178 mV, and the encapsulation efficiency was 87.64 ± 8.622%. All physicochemical values of KC2-ChrimsonR LNPs are shown in Table 1 and DLS histogram plots (Supplementary Figure S3).

### 3.2. Neuron Morphology

We tested KC2-ChrimsonR LNPs at plasmid concentrations of 0.25, 0.50, 0.75, 1, 1.25, 1.50, 1.75, and 2 µg in cells either on 7–11 DIV or 21–28 DIV. After 24 h, all tested concentrations successfully delivered the ChrimsonR plasmid (Supplementary Figure S4), as we observed in multiple neurons the expression of the tdTomato reporter. However, the morphology of KC2-ChrimsonR-transfected cells appeared distinct compared to neurons treated with the commercially available reagent lipofectamine. To quantify and characterize these differences, we measured (1) the number of dendrites, (2) the branching points, and (3) the total length of all dendrites.

**Figure 2:**
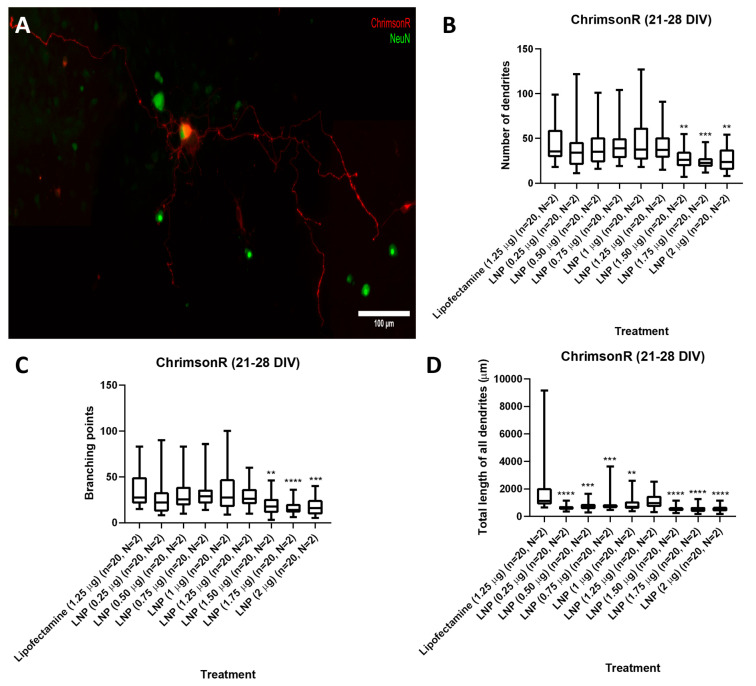
Morphological analysis in 21–28 DIV neurons transfected with KC2-ChrimsonR LNPs. (**A**) Morphological aspect of a cortical neuron treated with KC2-ChrimsonR LNPs (scale bar: 100 µm). 21–28 DIV rat cortical neurons treated with KC2-ChrimsonR LNPs showed reduction in morphological parameters as number of dendrites (**B**), branching points (**C**), and total length of all dendrites (**D**) compared with the lipofectamine treatment (Mann–Whitney test, ** *p* < 0.01, *** *p* < 0.001, **** *p* < 0.0001, *n* = number of cells, N = number of cultures).

Results showed, in 21–28 DIV transfected neurons (Figure 2A), that there was no statistical difference in the number of dendrites parameter between the lipofectamine control and 0.25 to 1.25 µg KC2-ChrimsonR treatments, except with treatments 1.50 µg (*p*-value = 0.0045 **), 1.75 µg (*p*-value = 0.0002 ***), and 2 µg (*p*-value = 0.0052 **) (Figure 2B). A similar outcome was observed with the branching points parameter, with no statistical difference between the lipofectamine control and 0.25 to 1.25 µg KC2-ChrimsonR treatments, except with treatments 1.50 µg (*p*-value = 0.0024 **), 1.75 µg (*p*-value < 0.0001 ****), and 2 µg (*p*-value = 0.0005 **) (Figure 2C). However, a significant reduction was observed in the total length of all dendrites between the lipofectamine control and all the KC2-ChrimsonR-treated neurons, except for the 1.25 µg KC2-ChrimsonR treatment, which had no statistical significance compared to the lipofectamine control (*p*-value = 0.0965) (Figure 2D). All *p*-values can be found in Supplementary Table S1.

Regarding the morphological parameters observed on 7–11 DIV transfected cells (Supplementary Figure S5A), differences were observed in comparison to those observed in 21–28 DIV transfected neurons. In the number of dendrites, the most notable difference was with 1.25 µg of encapsulated ChrimsonR plasmid. Here, neurons had a higher number of dendrites when compared to the lipofectamine control (*p*-value = 0.0022 **) (Supplementary Figure S5B). The rest of the KC2-ChrimsonR-treated neurons showed a statistically significant reduction in the number of dendrites compared to the lipofectamine control, except the 0.25 µg KC2-ChrimsonR-treated neurons (*p*-value = 0.0795). A similar trend was observed in branching points, in which the 1.25 µg treatment showed an increase (*p*-value = 0.0213 *) (Supplementary Figure S5C). However, all other treatments showed a significant reduction, with the 0.25 µg dose treatment being the least significant (*p*-value = 0.0169 *). In total length of all dendrites, a robust statistical reduction was observed in all KC2-ChrimsonR treatments (*p*-value < 0.0001 ****), except for the 1.25 µg KC2-ChrimsonR treatment, with no statistical difference compared to the lipofectamine control (*p*-value = 0.6980) (Supplementary Figure S5D). All *p*-values can be found in Supplementary Table S2.

### 3.3. Transfection Efficiency

**Figure 3:**
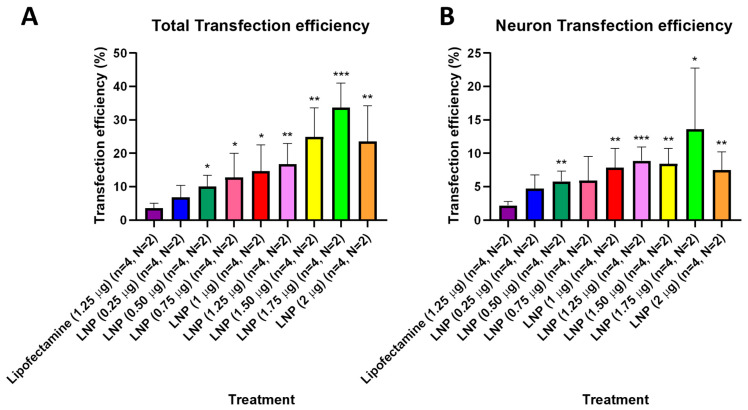
Transfection efficiency of total cells and neurons of KC2-ChrimsonR LNPs in 21–28 DIV. 21–28 DIV cultures treated with KC2-ChrimsonR LNPs showed increased transfection efficiency compared to lipofectamine controls in total transfection (**A**) and neuron transfection (**B**) (multiple t-test, * *p* < 0.05, ** *p* < 0.01, *** *p* < 0.001, *n* = number of coverslips, N = number of cultures).

After observing that KC2-ChrimsonR LNPs could effectively deliver the ChrimsonR plasmid in neurons, we tried to determine what percentage of cells expressed the ChrimsonR plasmid in brain cortical primary cultures on 7–11 DIV and 21–28 DIV (Supplementary Figure S6). In 21–28 DIV cultures, the percentage of total transfection efficiency was statistically significantly higher than lipofectamine control (3.55%) in all KC2-ChrimsonR dose treatments, except at 0.25 µg (6.88%, *p*-value = 0.1356) (Figure 3A). Moreover, the percentage of total transfection efficiency increased as the concentration of KC2-ChrimsonR treatments increased, with the highest values in 1.25 µg (16.83%, *p*-value = 0.0056 **), 1.50 µg (24.93%, *p*-value = 0.0029 **), 1.75 µg (33.70%, *p*-value = 0.0001 ***), and 2 µg (23.60%, *p*-value = 0.0095 **) KC2-ChrimsonR treatments. When only considering neuron transfection efficiency, the percentage of all KC2-ChrimsonR treatments was statistically significantly higher than lipofectamine control (2.20%), except for 0.25 µg (4.72%, *p*-value = 0.0572) and 0.75 µg (5.95%, *p*-value = 0.0852) KC2-ChrimsonR treatments. The highest efficiency observed was with the 1.25 µg (8.89%, *p*-value = 0.0007 ***) treatment, and the lowest statistical difference was with the 1.75 µg (13.61%, *p*-value = 0.0479 *) treatment (Figure 3B). All *p*-values are provided in Supplementary Table S3.

In respect of the transfection efficiency of 7–11 DIV cultures, all the KC2-ChrimsonR treatments showed percentages that were statistically significantly higher than the lipofectamine control (3.55%), with the highest statistical significance in 1.50 µg (56.74%, *p*-value < 0.0001 ****) and 1.75 µg (58.33%, *p*-value < 0.0001 ****) (Supplementary Figure S7A). Similarly, the same trend was observed in neuron transfection efficiency with 1.50 µg (34.11%, *p*-value < 0.0001 ****) and 1.75 µg (36.38%, *p*-value < 0.0001 ****) KC2-ChrimsonR treatments (Supplementary Figure S7B). All *p*-values are provided in Supplementary Table S4.

### 3.4. Neuron Electrophysiology

After analyzing both the morphological parameters and the transfection efficiency of the different KC2-ChrimsonR treatments, we chose 1.25 µg as the optimal treatment for measuring neuron electrophysiology. Although some morphological changes were observed after KC2-ChrimsonR treatment compared to lipofectamine control, we wanted to observe if these changes were accompanied by electrophysiological changes. To find out, we performed whole-cell patch clamp recordings on neurons transfected after 21–28 DIV with KC2-ChrimsonR LNPs. Both KC2-ChrimsonR LNPs and lipofectamine-transfected neurons were photostimulated with 2 pulses (5 ms and 1 s interpulse) of amber light (590 nm).

**Figure 4:**
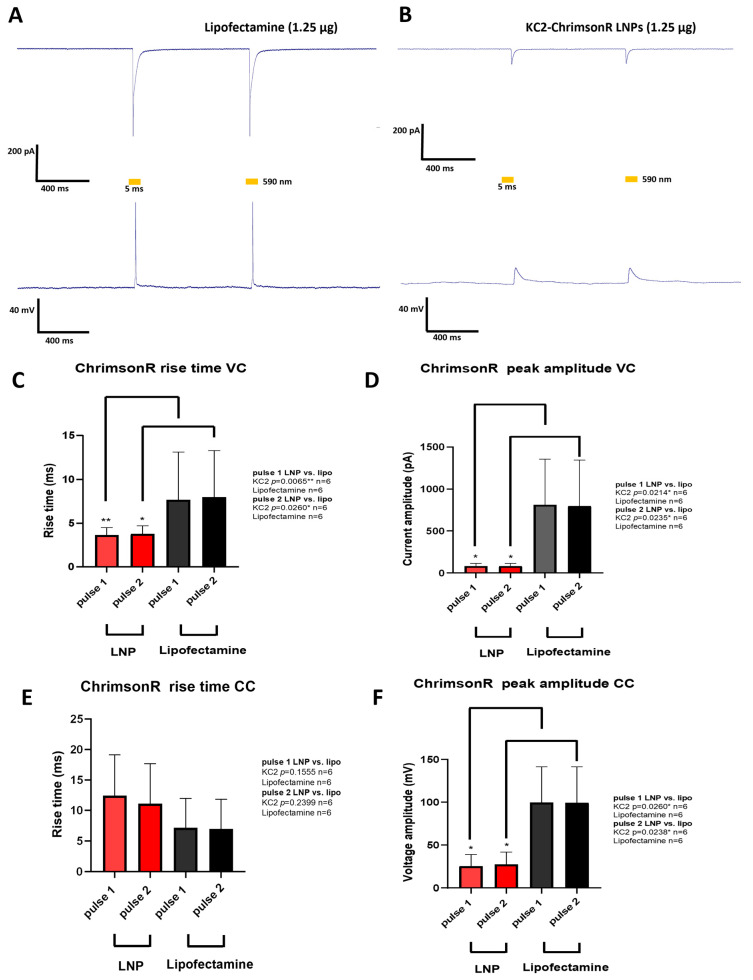
Electrophysiological changes induced by KC2-ChrimsonR LNPs. (**A**) Representative photostimulation of a rat cortical neuron DIV 28 transfected with lipofectamine (1.25 µg) expressing ChrimsonR, showing AP firing. (**B**) Representative photostimulation of a rat cortical neuron DIV28 transfected with KC2-ChrimsonR LNPs (1.25 µg), showing membrane depolarization without AP firing. Voltage-clamp (VC, top) and current-clamp (CC, bottom) recordings were obtained while cells were photostimulated with two pulses of 5 ms (590 nm) with a 1 s interspace. (**C**–**F**) Comparative analysis between lipofectamine and KC2-ChrimsonR LNPs in rise time and peak amplitude electrophysiological parameters in each light pulse (Mann–Whitney test (VC rise time and CC peak amplitude) and Welch’s test (VC peak amplitude and CC rise time); * *p* < 0.05, ** *p* < 0.01. Graphs bars are expressed as mean ± SD.

Cells transfected with lipofectamine (*n* = 6) showed strong inward currents and light-driven action potentials (Figure 4A), but this was not the case for KC2-ChrimsonR-treated neurons (*n* = 6), which showed small inward currents and depolarizations but not action potentials (Figure 4B). After performing voltage-clamp (VC) recordings, we observed faster rise times in KC2-ChrimsonR-treated neurons compared to lipofectamine controls in pulse 1 (*p*-value = 0.0065 **, Mann–Whitney test) and pulse 2 (*p*-value = 0.0260 *, Mann–Whitney test) (Figure 4C), and peak amplitudes were significantly smaller in pulse 1 (*p*-value = 0.0214 *, Welch’s test) and pulse 2 (*p*-value = 0.0235 *, Welch’s test) (Figure 4D).

When current-clamp (CC) recordings were performed, we observed no statistically significant difference in the rise times in KC2-ChrimsonR-treated neurons compared to lipofectamine-treated neurons in pulse 1 (*p*-value = 0.1555, Welch’s test) and pulse 2 (*p*-value = 0.2399, Welch’s test) (Figure 4E). On the other hand, when measuring peak amplitudes, we observed significantly smaller amplitudes for pulse 1 (*p*-value = 0.0260 *, Mann–Whitney test) and pulse 2 (*p*-value = 0.0238 *, Mann–Whitney test) (Figure 4F).

### 3.5. Cell Viability

**Figure 5:**
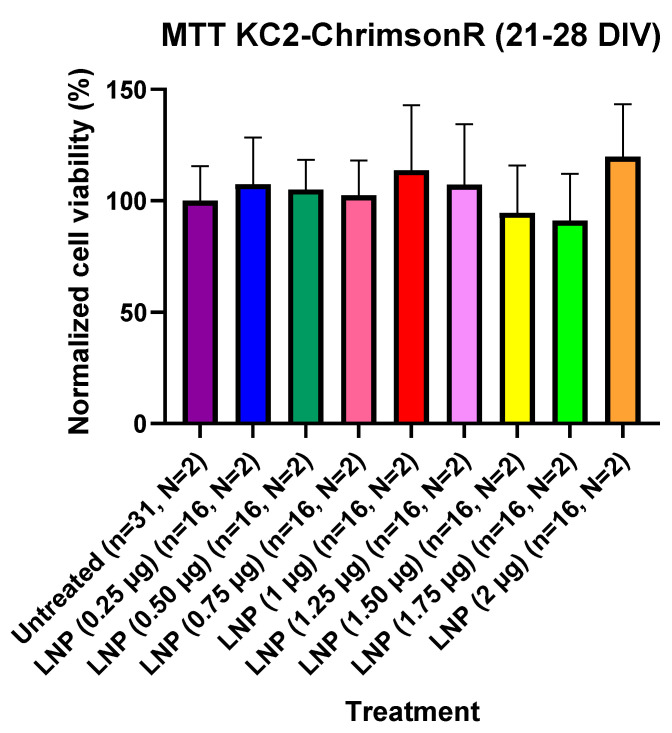
Cell viability using KC2-ChrimsonR LNPs in cortical neurons at 21–28 DIV. MTT assays performed in 21–28 DIV rat cortical neurons at 24 showed no reduction in cell viability values at any concentration compared with untreated controls (Mann–Whitney test, *n* = number of wells, N = number of cultures). Graph bars are expressed as mean ± SD.

For assessing if KC2-ChrimsonR LNPs could affect cell viability, we performed MTT cell viability experiments in 21–28 DIV cells and in 7–11 DIV cells. Cells were seeded in 96-well plates, and we compared KC2-ChrimsonR-treated wells. For 21–28 DIV cells, we transfected wells and waited 24 h before measuring cell viability after MTT addition. After waiting 24 h and measuring MTT absorbance values after MTT addition, we observed that there was not a significant difference in MTT absorbance values in any of the treatments with KC2-ChrimsonR LNPs compared to untreated controls, indicating that KC2-ChrimsonR LNPs do not affect, at any of the tested concentrations, the cell viability of primary cortical cultures after 24 h (Figure 5). All *p*-values are provided in Supplementary Table S5.

However, when analyzing the cell viability in 7–11 DIV cells with KC2-ChrimsonR LNPs, we observed some differences compared to 21–28 DIV cells. Even though there was not a significant reduction in MTT absorbance values compared to untreated cells at 0.25 µg (*p*-value = 0.3629), 0.50 µg (*p*-value = 0.6529), 0.75 µg (*p*-value = 0.1240), and 1 µg (*p*-value = 0.0723) concentrations, we observed statistically higher cell viability values at 1.25 µg (*p*-value = 0.0123 *), 1.25 µg (*p*-value = 0.0123 *), 1.50 µg (*p*-value = 0.0169 *), 1.75 µg (*p*-value = 0.0026 **), and 2 µg (*p*-value = 0.0007 ***) concentrations (Supplementary Figure S8A).

To check if this trend happened over time, we performed similar experiments in 7–11 DIV cells, but incubating 48 h and 96 h after transfection with KC2-ChrimsonR LNPs. When checking the cell viability values of KC2-ChrimsonR-treated wells and untreated wells after 48 h incubation, we observed no statistical differences among treatments (Supplementary Figure S8B). Nevertheless, when checking MTT absorbance values after 96 h incubation, we observed no statistical differences between untreated cells and KC2-ChrimsonR LNPs-treated cells at concentrations 0.25 µg (*p*-value = 0.4068), and 0.75 µg (*p*-value = 0.0934), but we observed a statistically significant decrease in MTT absorbance values at concentrations 0.50 µg (*p*-value = 0.0384 *), 1 µg (*p*-value = 0.0242 *), 1.25 µg (*p*-value = 0.0021 **), 1.50 µg (*p*-value = 0.0006 ***), 1.75 µg (*p*-value = 0.0003 ***), and 2 µg (*p*-value = 0.0063 **) (Supplementary Figure S8C). All *p*-values can be found in Supplementary Table S6.

## 4. Discussion

Here we have used, for the first time, LNPs for optogenetic delivery purposes and compared transfected cells with lipofectamine. KC2-ChrimsonR LNPs demonstrated the ability to deliver the optogenetic plasmid ChrimsonR in a wide range of concentrations. Fluorescent reporter expression confirmed the localization of the optogenetic protein to the cell membrane, and no significant changes in the neuron morphology in most KC2-ChrimsonR treatments were observed. However, in 21–28 DIV neurons treated with concentrations higher than 1.25 µg, we observed a reduction in the number of dendrites, branching points, and total length of all dendrites compared to lipofectamine-treated cells. This indicates that, probably, concentrations superior to 1.25 µg can be toxic to neurons.

Regarding the morphological changes of 7–11 DIV neurons, the trends were somewhat different. Here, a reduction in the number of dendrites and branching points was observed in almost every concentration. Using a 1.25 µg concentration, we observed an increase in the number of dendrites and branching points compared to lipofectamine. In the total length of all dendrites there was a robust reduction in all the parameters at every concentration, with the only exception being the 1.25 µg concentration. These differences indicate that younger cells might be more sensitive to KC2-ChrimsonR LNPs compared to more mature and electrophysiologically active neurons.

One possible explanation for these changes could be related to the concentration of cationic lipids in the LNPs, as cationic lipids have been reported to have toxic effects on cells, which include reactive oxygen species (ROS) formation [42,43,44]. In previous studies, the increment of ROS has been associated with negative regulation of dendritic arbor size [45,46], which could explain the changes observed in the dendritic arbors of neurons treated with KC2-ChrimsonR LNPs. This problem could be addressed by using several strategies, such as using antioxidants [47], biodegradable cationic lipids [48], and cationic amphiphiles [49]. Solving this problem could be helpful for LNP-mediated neuronal transfection delivery, as LNP delivery has been tested previously in different types of neurons [50,51], making optimized LNPs good candidates for optogenetic neuronal delivery.

In 21–28 DIV cultures, all KC2-ChrimsonR LNP concentrations (except 0.25 µg) achieved higher transfection efficiency than the 1.25 µg lipofectamine condition. Importantly, this advantage was maintained even at LNP doses below 1.25 µg. A similar trend was observed when quantifying the number of neurons transfected at each LNP concentration relative to controls. When checking the total transfection efficiency of 7–11 DIV cells, we observe that the differences in percentage of transfection were bigger than the ones observed in 21–28 DIV cells, with a similar trend observed when measuring only the transfected neurons. Unlike 21–28 DIV cells, all the KC2-ChrimsonR treatments widely surpassed the lipofectamine treatment in transfection efficiency. These results may reflect the higher cell density typically found in younger cortical primary cultures and/or increased susceptibility of younger cells to internalizing genetic material compared to older cells.

Unlike lipofectamine-treated neurons, KC2-ChrimsonR-treated neurons show reduced inward currents and depolarization with no action potential firing. The depolarization with no action potential firing indicates that the ChrimsonR channels open when photostimulated, but this is not enough to reach the threshold and fire action potentials.

Also, the rise time in VC recordings of KC2-ChrimsonR neurons is significantly smaller than lipofectamine neurons, which is probably because of the smaller current elicited after photostimulation, taking less time to reach the peak. This significant shortcoming for a therapeutic application of this LNP formulation is probably a consequence of the reduction in dendritic arbors of neurons treated with these LNPs, as the reduction in dendritic arbors produces reduced synaptic currents [52]. Additionally, the ROS formation produced by LNP transfection may influence the electrophysiology of neurons by reducing the amplitude and frequency of electrophysiological signals [53], or by altering mitochondrial components, which ultimately leads to the reduction in ATP availability to maintain resting membrane potential and ionic gradients, making neurons less able to fire reliably [54]. These conditions emphasize the need to use strategies that can reduce the formation of ROS.

In our laboratory, we have previously characterized the electrophysiological responses of cortical neurons transfected with niosomes containing CAG-ChrimsonR plasmids, observing similar outcomes [55]. However, the differences between KC2-ChrimsonR-transfected neurons (especially in peak amplitudes) are smaller than the ones observed in niosome-transfected neurons, which could be seen as an improvement in electrophysiological properties. Even though there is room for improvement, this is the first time that ChrimsonR has been delivered with LNPs into neurons, as well as the first functional characterization of neuron responses with ChrimsonR under these circumstances. Additionally, we have previously achieved optogenetic delivery of ChR2 with magnetic nanoparticles (although with no functional characterization) in rat visual cortex [56], and delivery with niosomes of VEGF plasmids in mouse brain to promote brain angiogenesis in order to treat central nervous system (CNS) diseases [57].

Cell viability results reveal that KC2-ChrimsonR did not affect the MTT absorbance of 21–28 DIV cortical cultures, which is most likely due to the presence of PEG in these LNPs, as PEG has been proven to enhance the biocompatibility of lipid-based nanoparticles [58,59]. However, cell viability results in 7–11 DIV cells showed a significant increase in their MTT absorbance values compared to untreated controls, suggesting altered metabolic activity of neurons from younger cultures following LNP internalization [60], but the exact mechanisms of this phenomenon are yet to be discovered. To check if these changes persisted over time, we incubated the cells with KC2-ChrimsonR nanoparticles for 48 h and 96 h. At 48 h incubation, there was no statistical difference in the MTT absorbance values of untreated and KC2-ChrimsonR-treated cells. Nevertheless, after 96 h of incubation, we observed a significant decrease in MTT absorbance values, especially at high KC2-ChrimsonR concentrations. This outcome suggests that an incubation time superior to 96 h could be toxic, especially at high concentrations. This could raise concerns about the harm that these LNPs could cause in animal models, but it must be taken into account that transfected primary cultures have no physiological barriers, bypassing body biodistribution and clearance mechanisms observed in animal models injected with LNPs [61,62]. Accordingly, this potential for longer-term toxicity should be carefully evaluated in future in vivo studies with extended observation periods.

An important limitation of this study is the absence of a direct comparison with empty LNPs, which would help to further distinguish between effects arising from the genetic payload and any potential non-specific effects of the delivery vehicle. Although earlier experiments using this composition did not reveal detectable functional alterations in neurons treated with empty LNPs, dedicated studies incorporating vehicle-only controls will be necessary to conclusively rule out such effects.

## 5. Conclusions

Our results show, for the first time, the use of LNPs for optogenetic delivery with functional activity, suggesting that LNPs could be good candidates for transfecting optogenetic genes with appropriate formulation improvement. These LNPs demonstrate an improvement on previously reported niosomes in terms of transfection efficiency, overall morphology, and electrophysiology. Nevertheless, these results also suggest that there is scope for improvement, as we observed some morphological and electrophysiological changes that negatively affect neurons transfected with LNPs. Therefore, engineering new and optimized formulations is vital for the development of advanced optogenetics applications based on this type of non-viral vector.

## Figures and Tables

**Table 1:** Physicochemical parameters of KC2-ChrimsonR LNPs.

Size (nm)	PDI	Zeta Potential (mV)	Encapsulation Efficiency (%)
82.295 ± 7.687	0.138 ± 0.053	−7.311 ± 7.178	87.64 ± 8.622

## Data Availability

Data is contained within the article and the Supplementary Material.

## Supplementary Materials

The following supporting information can be downloaded at https://www.mdpi.com/article/10.3390/pharmaceutics18010004/s1. Figure S1: Schematic representation of the pCAG-ChrimsonR-tdTomato plasmid (7301 bp) used for optogenetic delivery; Figure S2: TEM image of KC2-ChrimsonR LNPs; Figure S3: Size distribution DLS histogram plots of KC2-ChrimsonR LNPs in terms of intensity (A) and volume (B) percentages; Figure S4: Representative images of neurons transfected with different dosages of KC2-ChrimsonR LNPs; Figure S5: Morphological analysis in 7–11 DIV neurons transfected with KC2-ChrimsonR LNPs; Table S1: *p*-values of 21–28 DIV neurons treated with KC2-ChrimsonR LNPs compared with lipofectamine controls in morphological analysis; Table S2: *p*-values of 7–11 DIV neurons treated with KC2-ChrimsonR LNPs compared with lipofectamine controls in morphological analysis; Figure S6: Representative images of ChrimsonR expressing neurons for transfection efficiency quantification; Figure S7: Transfection efficiency of total cells and neurons of KC2-ChrimsonR LNPs in 7–11 DIV cells; Table S3: *p*-values of 21–28 DIV neurons treated with KC2-ChrimsonR LNPs compared with lipofectamine controls in transfection efficiency; Table S4 *p*-values of 7–11 DIV neurons treated with KC2-ChrimsonR LNPs compared with lipofectamine controls in transfection efficiency; Table S5: *p*-values of 21–18 DIV neurons with KC2-ChrimsonR LNPs compared with untreated cells in MTT assays; Figure S8: Cell viability using KC2-ChrimsonR LNPs in 7–11 DIV cells; Table S6: *p*-values of 7–11 DIV neurons treated with KC2-ChrimsonR LNPs compared with untreated cells in MTT assays.
