# Supplementary material for: Evaluation of Lipid Nanoparticles as Vehicles for Optogenetic Delivery in Primary Cortical Neurons
Source: Pharmaceutics. 2025 Dec 19;18(1):4. doi: 10.3390/pharmaceutics18010004 (PMC12845084; doi:10.3390/pharmaceutics18010004)
Supplement: Supplementary file 1 [file pharmaceutics-18-00004-s001.zip › pharmaceutics-4011161-supplementary.pdf]

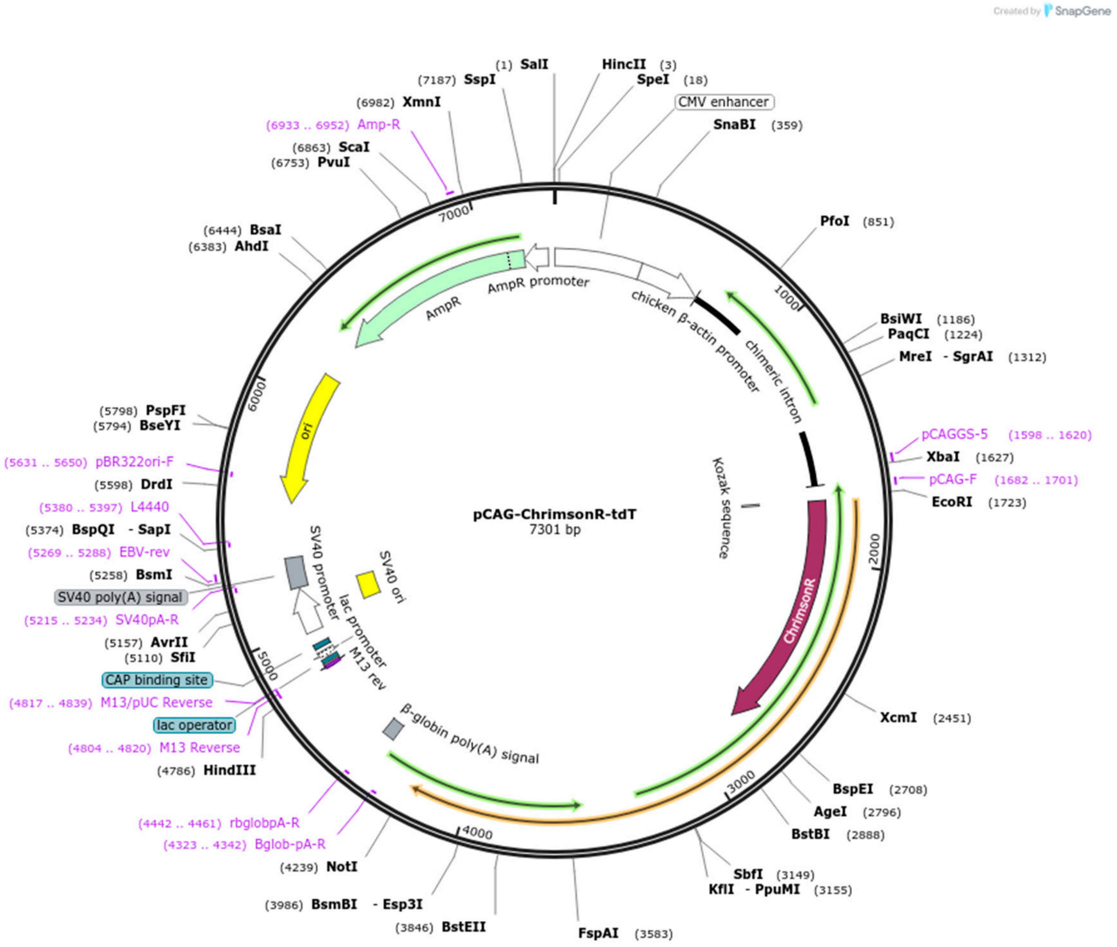

Supplementary Figure S1. Schematic representation of the pCAG-ChrimsonR-tdTomato plasmid (7301 bp) used for optogenetic delivery. The construct contains the hybrid CAG promoter (CMV enhancer/chicken β-actin promoter), followed by a chimeric intron and the ChrimsonR-tdTomato coding sequence. The plasmid also includes a β-globin poly(A) signal for transcript termination and an SV40 ori for replication in mammalian cells. Bacterial propagation is supported by the Ampicilin resistance cassette and pBR322 origin of replication. Relevant restriction sites are indicated.

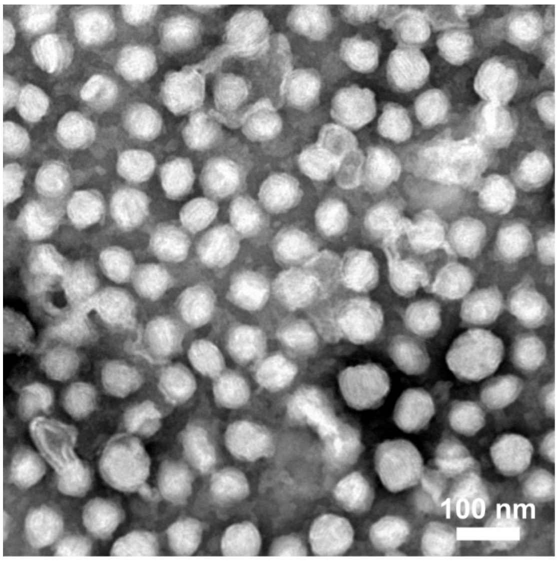

Supplementary Figure S2. TEM image of KC2-ChrimsonR LNPs. Scale bar: 100 nm.

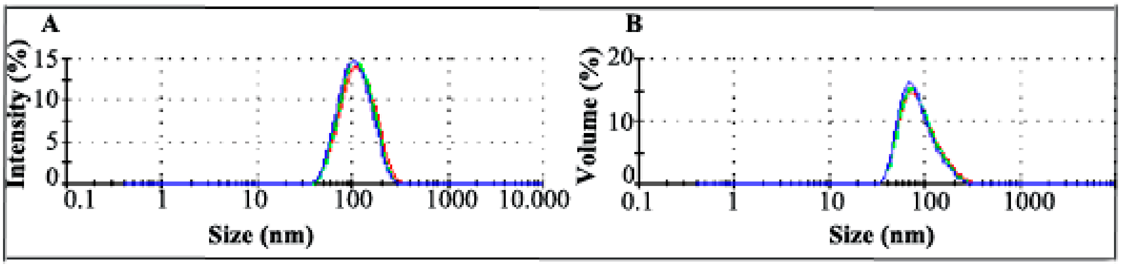

Supplementary Figure S3. Size distribution DLS histogram plots of KC2-ChrimsonR LNPs in terms of intensity (A) and volume (B) percentages.

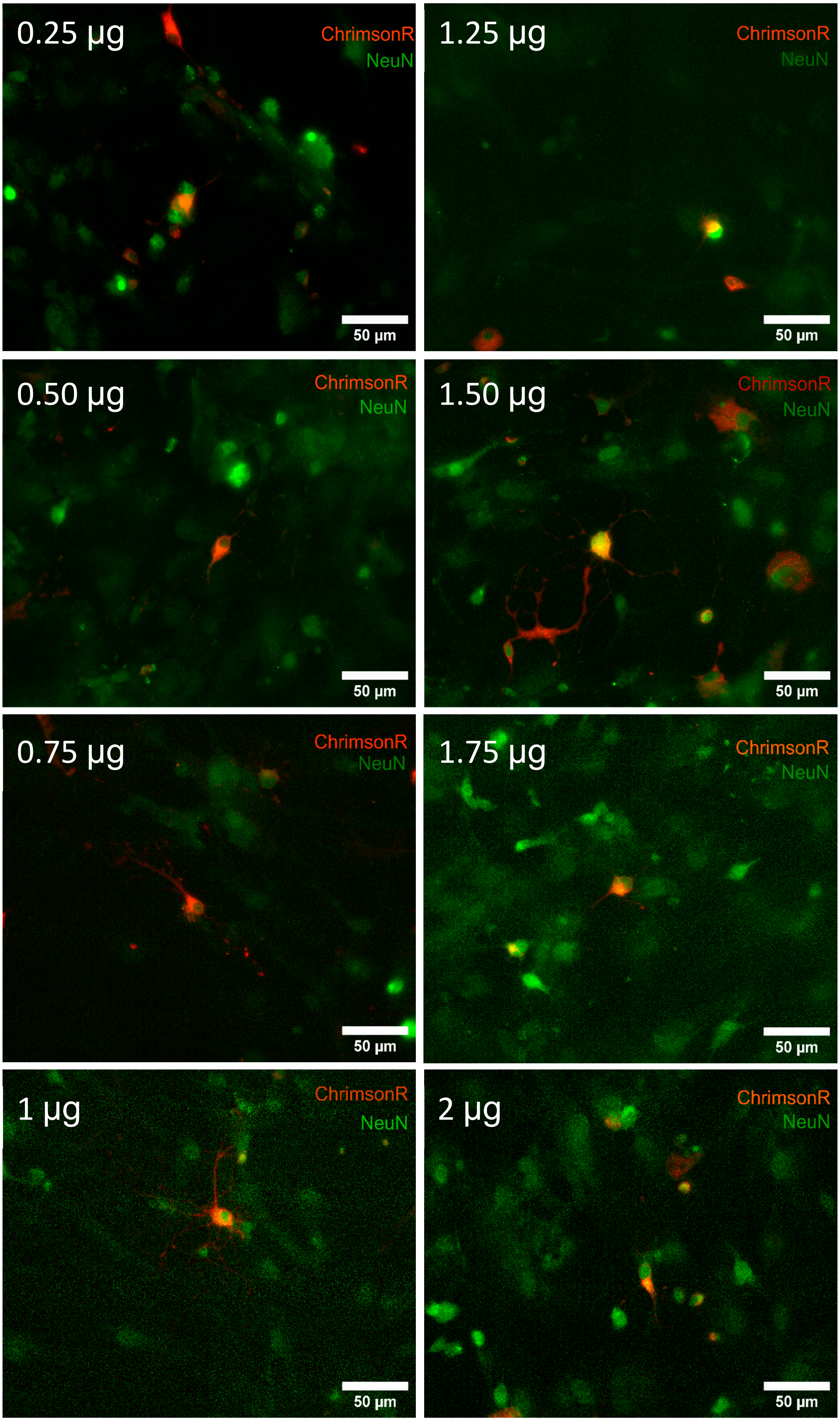

Supplementary Figure S4. Representative images of neurons transfected with different dosages of KC2-ChrimsonR LNPs. Scale bar: 50 µm.

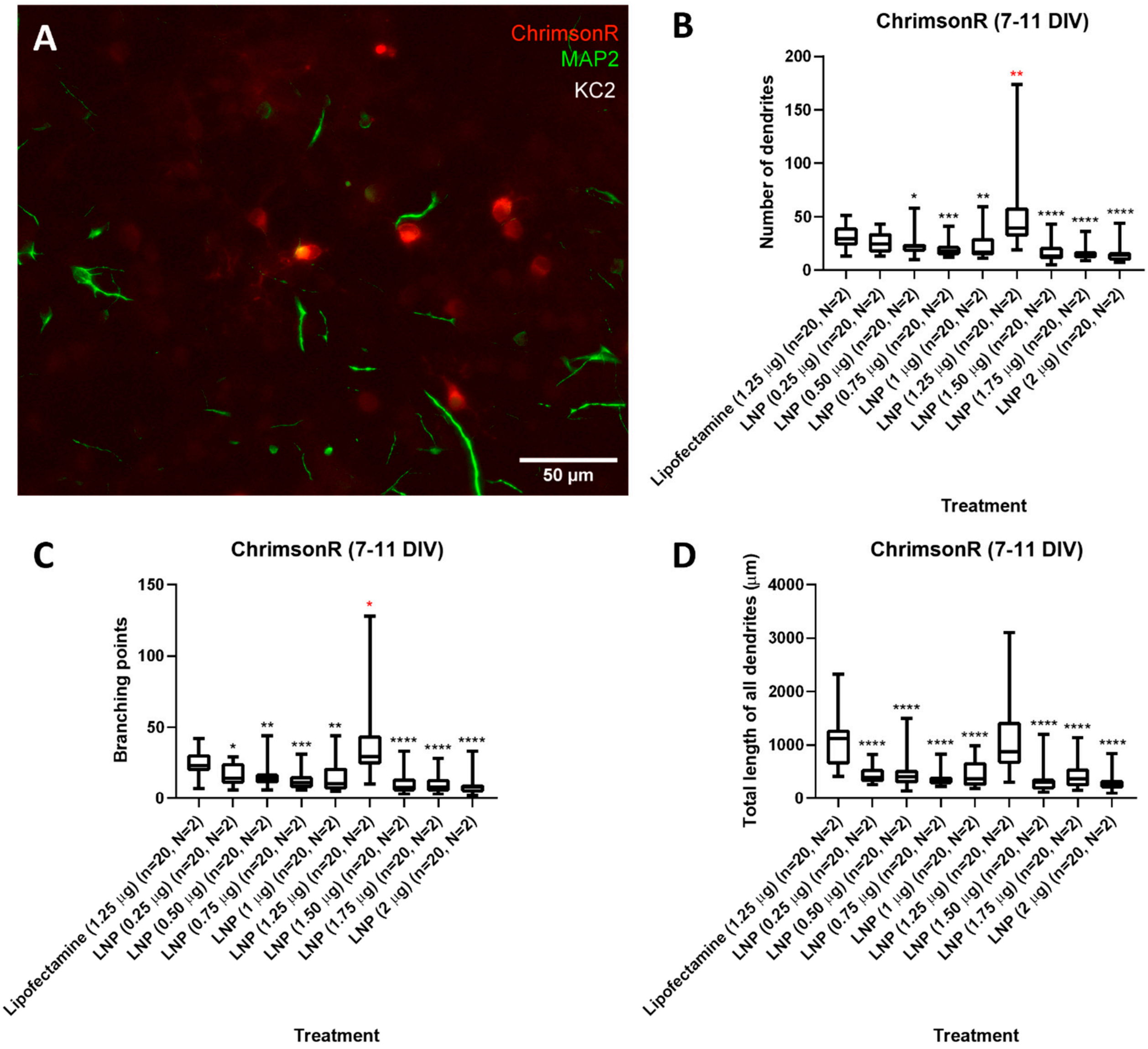

Supplementary Figure S5. Morphological analysis in 7-11 DIV neurons transfected with KC2-ChrimsonR LNPs (Scale bar: 50 µm). (A) Morphological aspect of 7-11 DIV cortical neurons treated with KC2-ChrimsonR LNPs. 7-11 DIV rat cortical neurons treated with KC2-ChrimsonR LNPs showed reduction in morphological parameters as number of dendrites (B) branching points (C), and total length of all dendrites (D) compared with the lipofectamine treatment (Mann-Whitney test, * $p < 0.05$ ** $p < 0.01$, *** $p < 0.001$, **** $p < 0.0001$, n = number of cells, N = number of cultures). Red *p*-values mean that there is statistical difference with groups with higher mean values than the untreated groups.

Morphology 21-28 DIV neurons	Number of dendrites	Branching points	Total length of all dendrites
	Lipofectamine (1.25 µg) (n=20, N=2)	Lipofectamine (1.25 µg) (n=20, N=2)	Lipofectamine (1.25 µg) (n=20, N=2)
**LNP (0.25 µg) (n=20, N=2)**	$p = 0.1845$	$p = 0.0772$	$p < 0.0001$ ****
**LNP (0.50 µg) (n=20, N=2**	$p = 0.5245$	$p = 0.4815$	$p = 0.0002$ ***
**LNP (0.75 µg) (n=20, N=2)**	$p = 0.9307$	$p = 0.6438$	$p = 0.0001$ ***
**LNP (1 µg) (n=20, N=2)**	$p = 0.8462$	$p = 0.6441$	$p = 0.0018$ **
**LNP (1.25 µg) (n=20, N=2)**	$p = 0.6732$	$p = 0.3506$	$p = 0.0965$
**LNP (1.50 µg) (n=20, N=2)**	$p = 0.0045$ **	$p = 0.0024$ **	$p < 0.0001$ ****
**LNP (1.75 µg) (n=20, N=2)**	$p = 0.0002$ ***	$p < 0.0001$ ****	$p < 0.0001$ ****
**LNP (2 µg) (n=20, N=2)**	$p = 0.0052$ **	$p = 0.0005$ ***	$p < 0.0001$ ****

Supplementary Table S1. *p*-values of 21-28 DIV neurons treated with KC2-ChrimsonR LNPs compared with lipofectamine controls in morphological analysis.

Morphology 7-11 DIV neurons	Number of dendrites	Branching points	Total length of all dendrites
	Lipofectamine (1.25 µg) (n=20, N=2)	Lipofectamine (1.25 µg) (n=20, N=2)	Lipofectamine (1.25 µg) (n=20, N=2)
**LNP (0.25 µg) (n=20, N=2)**	$p = 0.0795$	$p = 0.0169$ *	$p < 0.0001$ ****
**LNP (0.50 µg) (n=20, N=2**	$p = 0.0198$ *	$p = 0.0021$ **	$p < 0.0001$ ****
**LNP (0.75 µg) (n=20, N=2)**	$p = 0.0009$ ***	$p = 0.0002$ ***	$p < 0.0001$ ****
**LNP (1 µg) (n=20, N=2)**	$p = 0.0022$ **	$p = 0.0016$ **	$p < 0.0001$ ****
**LNP (1.25 µg) (n=20, N=2)**	$p = 0.0089$ **	$p = 0.0213$ *	$p = 0.6980$
**LNP (1.50 µg) (n=20, N=2)**	$p < 0.0001$ ****	$p < 0.0001$ ****	$p < 0.0001$ ****
**LNP (1.75 µg) (n=20, N=2)**	$p < 0.0001$ ****	$p < 0.0001$ ****	$p < 0.0001$ ****
**LNP (2 µg) (n=20, N=2)**	$p < 0.0001$ ****	$p < 0.0001$ ****	$p < 0.0001$ ****

Supplementary Table S2. *p*-values of 7-11 DIV neurons treated with KC2-ChrimsonR LNPs compared with lipofectamine controls in morphological analysis. Red p-values mean that there is statistical difference with groups with higher mean values than the untreated groups, while black p-values mean that there is statistical difference with groups with lower mean values than the lipofectamine control.

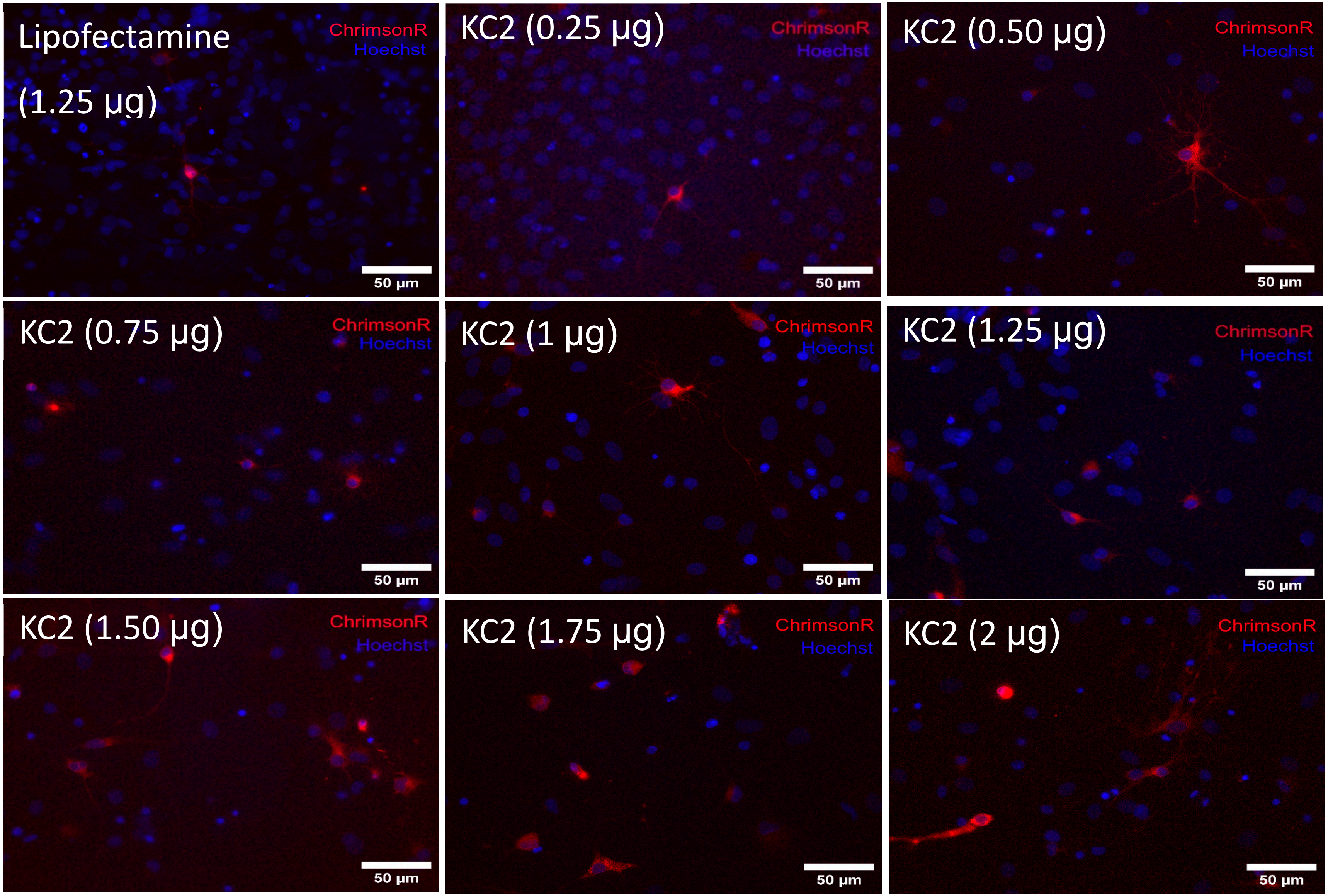

Supplementary Figure S6. Representative images of ChrimsonR expressing neurons for transfection efficiency quantification. Scale bar: 50 µm.

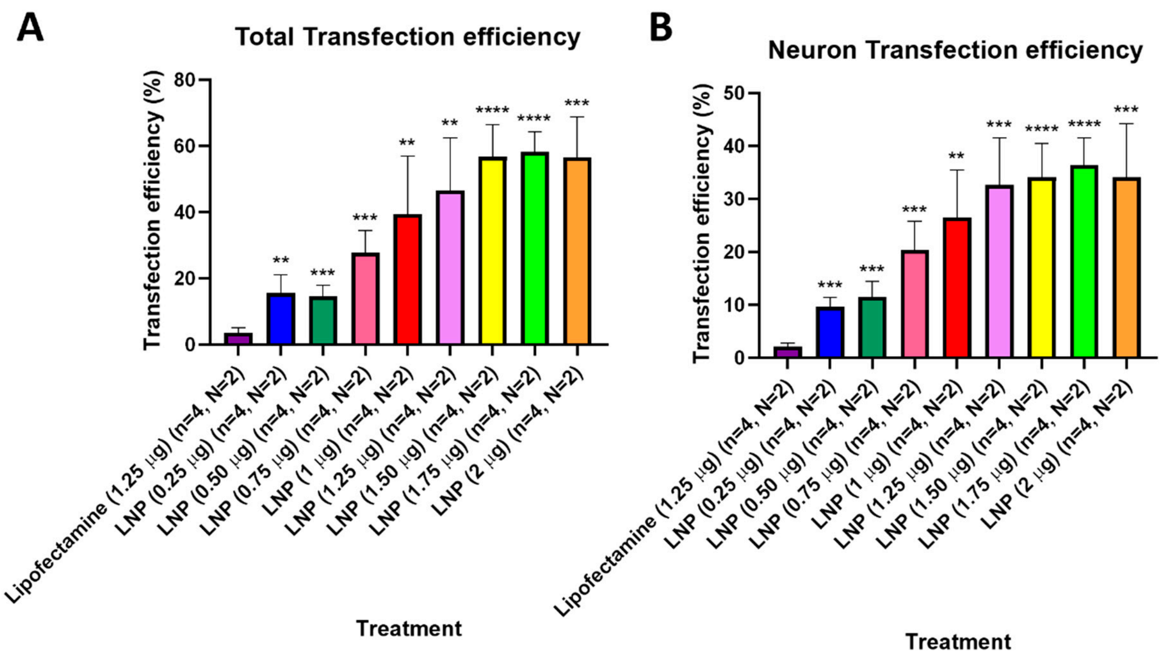

Supplementary Figure S7. Transfection efficiency of total cells and neurons of KC2-ChrimsonR LNPs in 7-11 DIV cells. 7-11 DIV rat cortical neurons treated with KC2-ChrimsonR LNPs showed increased transfection efficiency compared to lipofectamine controls in total transfection (A), and neuron transfection (B) (Multiple *t*-test, ** p <0.01, *** p <0.001, **** p <0.0001, n = number of coverslips, N = number of cultures).

Transfection efficiency 21-28 DIV neurons	Total transfection efficiency	Neuron transfection efficiency
	**Lipofectamine (1.25 µg) (n=4, N=2)**	**Lipofectamine (1.25 µg) (n=4, N=2)**
**LNP (0.25 µg) (n=4, N=2)**	*p* = 0.1356	*p* = 0.0572
**LNP (0.50 µg) (n=4, N=2**	*p* = 0.0124 *	*p* = 0.0046 **
**LNP (0.75 µg) (n=4, N=2)**	*p* = 0.0451 *	*p* = 0.0852
**LNP (1 µg) (n=4, N=2)**	*p* = 0.0301 *	*p* = 0.0081 **
**LNP (1.25 µg) (n=4, N=2)**	*p* = 0.0056 **	*p* = 0.0007 ***
**LNP (1.50 µg) (n=4, N=2)**	*p* = 0.0029 **	*p* = 0.0018 **
**LNP (1.75 µg) (n=4, N=2)**	*p* = 0.0001 ***	*p* = 0.0479 *
**LNP (2 µg) (n=4, N=2)**	*p* = 0.0095 **	*p* = 0.0083 **

Supplementary Table S3. *p*-values of 21-28 DIV neurons treated with KC2-ChrimsonR LNPs compared with lipofectamine controls in transfection efficiency.

Transfection efficiency 7-11 DIV neurons	Total transfection efficiency	Neuron transfection efficiency
	**Lipofectamine (1.25 µg) (n=4, N=2)**	**Lipofectamine (1.25 µg) (n=4, N=2)**
**LNP (0.25 µg) (n=4, N=2)**	*p* = 0.0060 **	*p* = 0.0002 ***
**LNP (0.50 µg) (n=4, N=2**	*p* = 0.0009 ***	*p* = 0.0008 ***
**LNP (0.75 µg) (n=4, N=2)**	*p* = 0.0003 ***	*p* = 0.0005 ***
**LNP (1 µg) (n=4, N=2)**	*p* = 0.0063 **	*p* = 0.0016 **
**LNP (1.25 µg) (n=4, N=2)**	*p* = 0.0016 **	*p* = 0.0004 ***
**LNP (1.50 µg) (n=4, N=2)**	*p* < 0.0001 ****	*p* < 0.0001 ****
**LNP (1.75 µg) (n=4, N=2)**	*p* < 0.0001 ****	*p* < 0.0001 ****
**LNP (2 µg) (n=4, N=2)**	*p* = 0.0001 ***	*p* = 0.0007 ***

Supplementary Table S4. *p*-values of 7-11 DIV neurons treated with KC2-ChrimsonR LNPs compared with lipofectamine controls in transfection efficiency.

**MTT**<br>**21-28 DIV cells**	**Untreated (n=31, N=2)**
	**24h**
**LNP (0.25 µg) (n=16, N=2)**	*p* = 0.7016
**LNP (0.50 µg) (n=16, N=2**	*p* = 0.4067
**LNP (0.75 µg) (n=16, N=2)**	*p* = 0.5822
**LNP (1 µg) (n=16, N=2)**	*p* = 0.8637
**LNP (1.25 µg) (n=16, N=2)**	*p* = 0.7432
**LNP (1.50 µg) (n=16, N=2)**	*p* = 0.1972
**LNP (1.75 µg) (n=16, N=2)**	*p* = 0.0678
**LNP (2 µg) (n=16, N=2)**	*p* = 0.1411

Supplementary Table S5. *p*-values of 21-28 DIV neurons treated with KC2-ChrimsonR LNPs compared with untreated cells in MTT assays.

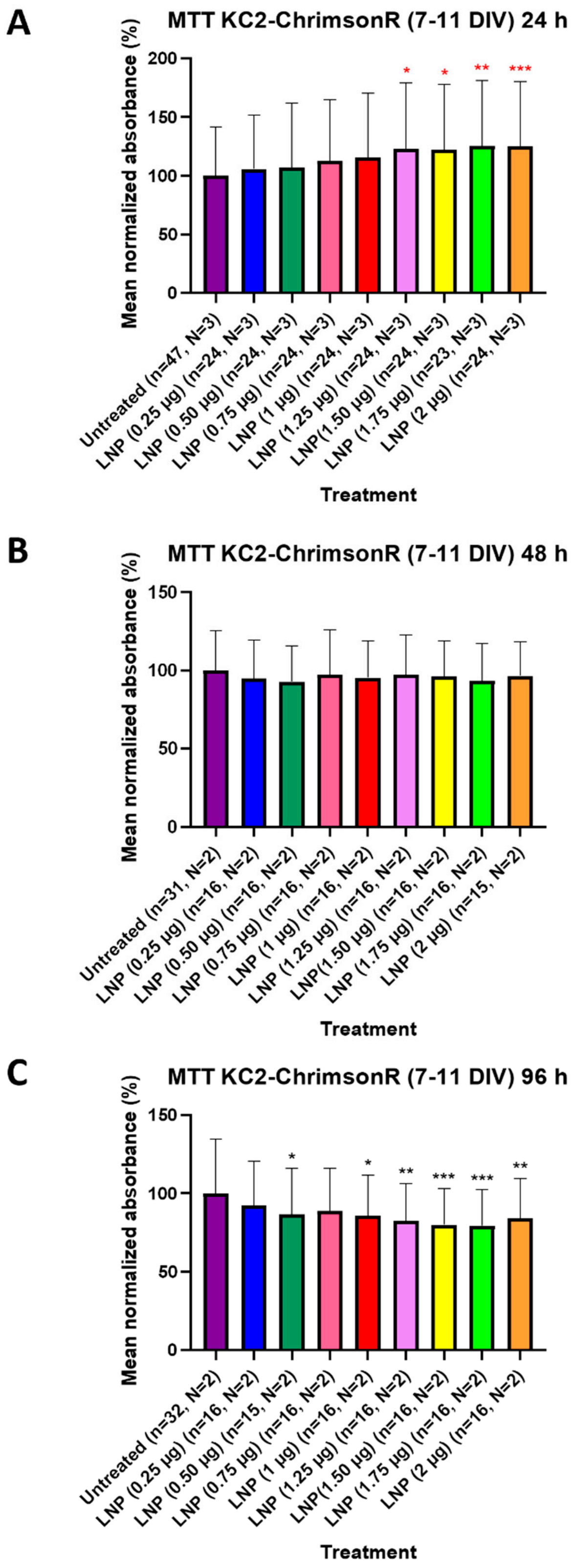

Supplementary Figure S8. Cell viability using KC2-ChrimsonR LNPs in 7-11 DIV cells. MTT assays performed in 7-11 DIV rat cortical neurons, showed increased MTT absorbance values at high KC2-ChrimsonR LNPs concentrations after 24 h incubation compared to untreated controls (A), no difference compared to untreated controls at 48 h incubation (B), and decreased MTT cell absorbance values at high KC2-ChrimsonR LNPs concentrations after 96 h incubation compared to untreated controls (C) (Mann-Whitney test, * p <0.05, ** p <0.01, *** p <0.001, n = number of wells, N = number of cultures). Graph bars are expressed as mean ± SD. Red p-values mean that there is statistical difference with groups with higher mean values than the untreated groups.

MTT 7-11 DIV neurons					
24h		48h		96h	
	Untreated (n=47, N=3)		Untreated (n=31, N=2)		Untreated (n=32, N=2)
LNP (0.25 µg) (n=24, N=3)	*p* = 0.3629	LNP (0.25 µg) (n=16, N=2)	*p* = 0.3945	LNP (0.25 µg) (n=16, N=2)	*p* = 0.4068
LNP (0.50 µg) (n=24, N=3)	*p* = 0.6529	LNP (0.50 µg) (n=16, N=2)	*p* = 0.1912	LNP (0.50 µg) (n=15, N=2)	*p* = 0.0384 *
LNP (0.75 µg) (n=24, N=3)	*p* = 0.1240	LNP (0.75 µg) (n=16, N=2)	*p* = 0.3822	LNP (0.75 µg) (n=16, N=2)	*p* = 0.0934
LNP (1 µg) (n=24, N=3)	*p* = 0.0723	LNP (1 µg) (n=16, N=2)	*p* = 0.4263	LNP (1 µg) (n=16, N=2)	*p* = 0.0242 *
LNP (1.25 µg) (n=24, N=3)	*p* = 0.0123 *	LNP (1.25 µg) (n=16, N=2)	*p* = 0.5226	LNP (1.25 µg) (n=16, N=2)	*p* = 0.0021 **
LNP (1.50 µg) (n=24, N=3)	*p* = 0.0169 *	LNP (1.50 µg) (n=16, N=2)	*p* = 0.4595	LNP (1.50 µg) (n=16, N=2)	*p* = 0.0006 ***
LNP (1.75 µg) (n=23, N=3)	*p* = 0.0026 **	LNP (1.75 µg) (n=16, N=2)	*p* = 0.2302	LNP (1.75 µg) (n=16, N=2)	*p* = 0.0003 ***
LNP (2 µg) (n=24, N=3)	*p* = 0.0007 ***	LNP (2 µg) (n=15, N=2)	*p* = 0.5818	LNP (2 µg) (n=16, N=2)	*p* = 0.0063 **

Supplementary Table S6. *p*-values of 7-11 DIV neurons treated with KC2-ChrimsonR LNPs compared with untreated cells in MTT assays. Red p-values mean that there is statistical difference with groups with higher mean values than the untreated groups, while black p-values mean that there is statistical difference with groups with lower mean values than the lipofectamine control.
